# Why choose *BJPsych Bulletin*?

**DOI:** 10.1192/bjb.2024.35

**Published:** 2024-06

**Authors:** Andrew Forrester

**Affiliations:** Cardiff University, Cardiff, UK

**Keywords:** *BJPsych Bulletin*, psychiatry, mental health, authors, readers

## Abstract

*BJPsych Bulletin* was first established as the *Bulletin of the Royal College of Psychiatrists* in 1977. Since then, it has extended its influence within the field, and it is now the go-to journal for practical clinical considerations in psychiatry, and mental health more widely. It stands together with the wider family of RCPsych journals – *BJPsych*, *BJPsych Advances*, *BJPsych Open* and *BJPsych International* – and offers a number of distinct advantages for readers and authors. I commend it to you.

*BJPsych Bulletin* has made a significant contribution to psychiatry since it was established first as the *Bulletin of the Royal College of Psychiatrists* in 1977. Over the years, it has undergone several name changes – *The Psychiatric Bulletin*, *The Psychiatrist*, *Psychiatric Bulletin* then *BJPsych Bulletin*, in 1988, 2010, 2014 and 2015 respectively. Yet despite these changes, the substance of the journal has continued to hold its own, making it a stalwart among psychiatric journals, a go-to journal for practical clinical considerations in psychiatry and mental health more widely, and a robust offer within the field.

I was delighted to take over as Editor in Chief of *BJPsych Bulletin* in late 2022. Since then, the dedication of the journal's Editorial Board has become increasingly apparent to me, and I thank each of the Board Members for the excellent work they do in keeping the journal on the road. I am also grateful to the wider administrative and publishing team for the often unseen, yet vital, work they do in ensuring that the journal's offer remains high quality – and to my immediate predecessor Norman Poole, for all the work he did in improving the journal and for the dedication he showed during our editorial handover.

*BJPsych Bulletin* is very fortunate in that it does not stand alone. Instead, it sits together with the family of RCPsych journal siblings – the *British Journal of Psychiatry* (*BJPsych*), of course, and also *BJPsych Advances*, *BJPsych Open* and *BJPsych International* (edited respectively by my colleagues Gin Malhi, Asit Biswas, Kenneth Kaufman and Marinos Kyriakopoulos). Working together, as we do, brings considerable advantages. This collaboration ensures that each of the *BJPsych* journals is individually strengthened and provides a substantial hinterland from which we collectively derive benefit.

As to *BJPsych Bulletin* itself, it is always open access and published bimonthly, and there are no charges for authors. This significant offer deliberately aligns with the goals of the Royal College of Psychiatrists (RCPsych): it improves and promotes article accessibility within the field, helps with the rapid dissemination of new information and supports psychiatric practice in the UK and internationally.

As with the other *BJPsych* journals, *BJPsych Bulletin* is published by Cambridge University Press, acting on behalf of the RCPsych, and it retains and promotes a focus on essential and practical reading within psychiatry. It has been, and remains, the go-to journal for a range of professionals within psychiatry, as well as for other multi-disciplinary and multi-agency contributors and readers. With a focus on a broad range of topics within psychiatry, contributions from all specialties and subspecialties within mental health are welcome. A deliberately wide range of article types is available for authors (e.g. original and review papers, commentaries, against-the-stream articles, cultural reflections, education and training, praxis, opinions, and special articles, to name just some). We welcome international manuscripts, offer first-view before print publication, article collections and special editions, and have a solid social media presence.

As we advance, *BJPsych Bulletin* seeks to retain its robust yet practical role in the field. In addition to publishing high-quality research, we will continue to challenge conventional thought and ideas, stimulate discussion and debate, and reflect on our current practices with sensitivity, keeping one eye on the history of our subject. In moving forward within psychiatry, we must consider and learn lessons from the past, where they exist.

I commend *BJPsych Bulletin* to you, whether you are an author, a reader or another interested party, and I look forward to its development in the coming years.

## Data Availability

Data availability is not applicable to this article as no new data were created or analysed in its preparation.

## About the author

**Andrew Forrester** is Professor of Forensic Psychiatry in the Division of Psychological Medicine and Clinical Neurosciences, School of Medicine, Cardiff University, Cardiff, UK, and a consultant forensic psychiatrist with Swansea Bay University Health Board and Oxleas NHS Foundation Trust, where he is also Director of Research.

